# Controlled Mechanical Ventilation in Critically Ill Patients and the Potential Role of Venous Bagging in Acute Kidney Injury

**DOI:** 10.3390/jcm13051504

**Published:** 2024-03-05

**Authors:** Mark E. Seubert, Marco Goeijenbier

**Affiliations:** 1 Department of Intensive Care, HagaZiekenhuis, 2725 NA Zoetermeer, The Netherlands; 2 Department of Intensive Care, Spaarne Gasthuis, 2035 RC Haarlem, The Netherlands; mgoeijenbier@spaarnegasthuis.nl; 3 Department of Intensive Care, Erasmus MC, 3015 CN Rotterdam, The Netherlands

**Keywords:** acute kidney injury (AKI), pressure support ventilation (PSV), systemic vascular resistance (SVR), mean systemic filling pressure (MSVP), ICU delirium, cerebral lymph congestion, venous congestion, venous bagging

## Abstract

A very low incidence of acute kidney injury (AKI) has been observed in COVID-19 patients purposefully treated with early pressure support ventilation (PSV) compared to those receiving mainly controlled ventilation. The prevention of subdiaphragmatic venous congestion through limited fluid intake and the lowering of intrathoracic pressure is a possible and attractive explanation for this observed phenomenon. Both venous congestion, or “venous bagging”, and a positive fluid balance correlate with the occurrence of AKI. The impact of PSV on venous return, in addition to the effects of limiting intravenous fluids, may, at least in part, explain this even more clearly when there is no primary kidney disease or the presence of nephrotoxins. Optimizing the patient–ventilator interaction in PSV is challenging, in part because of the need for the ongoing titration of sedatives and opioids. The known benefits include improved ventilation/perfusion matching and reduced ventilator time. Furthermore, conservative fluid management positively influences cognitive and psychiatric morbidities in ICU patients and survivors. Here, it is hypothesized that cranial lymphatic congestion in relation to a more positive intrathoracic pressure, i.e., in patients predominantly treated with controlled mechanical ventilation (CMV), is a contributing risk factor for ICU delirium. No studies have addressed the question of how PSV can limit AKI, nor are there studies providing high-level evidence relating controlled mechanical ventilation to AKI. For this perspective article, we discuss studies in the literature demonstrating the effects of venous congestion leading to AKI. We aim to shed light on early PSV as a preventive measure, especially for the development of AKI and ICU delirium and emphasize the need for further research in this domain.

## 1. Introduction

We aim to explain our hypothetical model of both the increased risk of AKI and possible increased risk of ICU delirium in relation to controlled mechanical ventilation from a physiological perspective.

In 1967, Moreno et al. demonstrated that when intrathoracic pressure decreases, the venous return increases [1]. Acute kidney injury (AKI) in severely ill patients, which is characterized by a sudden or rapid deterioration in kidney function, can result from a variety of factors. These factors include, but are not limited to, decreased blood flow to the kidneys, inflammation, and direct damage to kidney tissues (as outlined in Table 1). Medical education teaches that acute kidney failure can be categorized into three main types: prerenal causes, renal causes, and postrenal causes, with the latter primarily associated with obstructions in the urinary tract. However, we propose that intrathoracic pressure can also contribute to the regulation of venous pressure in postrenal obstructions. To illustrate this, we consider the chosen ventilation mode in conjunction with venous congestion or what we call “venous bagging”, which represents the cumulative “weight” exerted on the subdiaphragmatic veins. This combination adds complexity to the equation and adds a fourth category, postrenal venous causes, to the AKI classification.

The increased risk of AKI in ventilated patients remains insufficiently explained [2]. Firstly, hemodynamic instability may arise because of mechanical ventilation together with the need for sedation and opioids. This can lead to decreased sympathetic activity as well as decreased blood pressure, cardiac output, and systemic vascular resistance, and thus potentially decreased renal blood flow. Secondly, ventilator-induced lung injury (VILI) triggers inflammation and the release of proinflammatory mediators, which can provoke a systemic inflammatory response. This cascade of systemic inflammation might contribute to ventilator-induced kidney injury (VIKI). The extent to which venous congestion and proinflammatory substances play a role in this process has yet to be comprehensively understood.

Positive end-expiratory pressure (PEEP), an indispensable component of mechanical ventilation, serves to maintain lung recruitment and enhance oxygenation. High PEEP levels can potentially elevate intra-abdominal pressure through increased intrathoracic pressure with the inherent caudal movement of the diaphragm, especially when the patient is paralyzed or deeply sedated, and in this way can either further aggravate venous congestion and hamper renal perfusion or increase the risk of AKI. Although high PEEP levels have been shown to be related to a fivefold increase in the risk of AKI in a multivariate analysis of COVID-19 patients, other studies suggest only a relation with invasive mechanical ventilation [2,3,4].

Additionally, an inadequate fluid balance, such as fluid overload or dehydration, has been shown to contribute to the development of AKI in mechanically ventilated patients; this warrants careful consideration in terms of management.

Moreover, critical illness is often associated with additional risk factors, such as sepsis, hypotension due to other causes, and the use of nephrotoxic medications, which can further heighten susceptibility to AKI, particularly in patients undergoing mechanical ventilation. Therefore, a comprehensive understanding of how mechanical ventilation influences renal function is crucial in managing the risks and optimizing patient outcomes.

In our recent study, we observed a remarkably low occurrence of AKI in COVID-19 patients who were intentionally ventilated with early pressure support ventilation (PSV) compared to groups of similarly ill patients who were primarily ventilated using controlled mechanical ventilation (CMV). In the subset of patients receiving PSV for more than 80% of the time during the initial 200 h of ventilation, no AKI occurred [5]. Notably, due to the acute onset of COVID-19 and the overwhelming number of hospitalized patients, selection bias at admission in our Dutch hospitals, although unlikely, cannot be ruled out.

We propose that limiting subdiaphragmatic venous congestion through PSV is a critical factor contributing to this favorable outcome when combined with careful fluid management, i.e., limiting fluid administration and enhancing venous return by lowering intrathoracic (and pleural) pressure. This contrasts with the absence of intrathoracic pressure reduction in the controlled ventilation setting.

In accordance with the same pathophysiologic concept, we further hypothesize that a potentially lower incidence of delirium in patients predominantly being treated with PSV may be due to a similar mechanism. Non-assisted respiration, by itself, appears to be a driver of meningeal lymph flow, and lowering the intrathoracic pressure may thus contribute to the removal of ICU-related toxins [6,7,8]. This, along with the demonstrated decrease in intracranial pressure (ICP) when lowering intrathoracic pressure, and the potential relevance of maintaining an upright position to facilitate gravity, could possibly reduce the risk of ICU-acquired cognitive decline. Using fewer sedatives and opioids when applying supportive ventilation compared to CMV could potentially bias this outcome because of the separate known relationship between dosing and ICU delirium.

Investigating the underlying mechanisms and comparing the outcomes between controlled mechanical ventilation and predominantly supportive mechanical ventilation may yield valuable insights into how to optimize ventilation strategies, minimize the risk of AKI, and potentially mitigate the occurrence of delirium in critically ill patients. We suggest that the mode of ventilation should at least be considered in AKI research in critical care medicine.

## 2. Mechanical Ventilation and AKI

The risk of acute kidney injury (AKI) is markedly elevated in ventilated patients, and factors such as increased PEEP or tidal volume settings alone do not fully account for this correlation [2,9,10]. Other contributing factors include hypercapnia and permissive hypoxemia, which can potentially compromise renal blood flow, as well as cause biotrauma, and can negatively impact cardiac output [11]. However, we propose that, in addition, a combination of reduced venous return and fluid loading with the bagging of the venous capacitance contributes to the occurrence of AKI in ventilated patients.

Adopting an approach of conservative fluid administration and employing compensatory increases in vasopressors has shown promise in reducing the risk of AKI development [12]. It is worth noting that cumulative fluid balance is strongly linked to the occurrence and progression of AKI [13]. While a hindered arterial renal blood flow undoubtedly poses a risk for kidney injury, venous congestion has been found to be even more harmful than arterial hypoperfusion [14,15,16].

In the past, the deterioration of kidney function was mainly attributed to hypoperfusion. In heart failure, surprisingly, there seems to be no correlation, or even a paradoxical relationship, between pump failure and renal dysfunction [17]. The preservation of renal perfusion within necessary boundaries can be attributed to mechanisms such as tubuloglomerular feedback and sympathetic-driven myogenic vasoconstriction, although evaluating renal autoregulation in human subjects remains challenging [18].

Nonetheless, this does not mean we can overlook renal arterial hypoperfusion and concomitant hypotension in our ICU patients, especially when preexisting hypertension is an additional risk factor [19].

**Figure 1:**
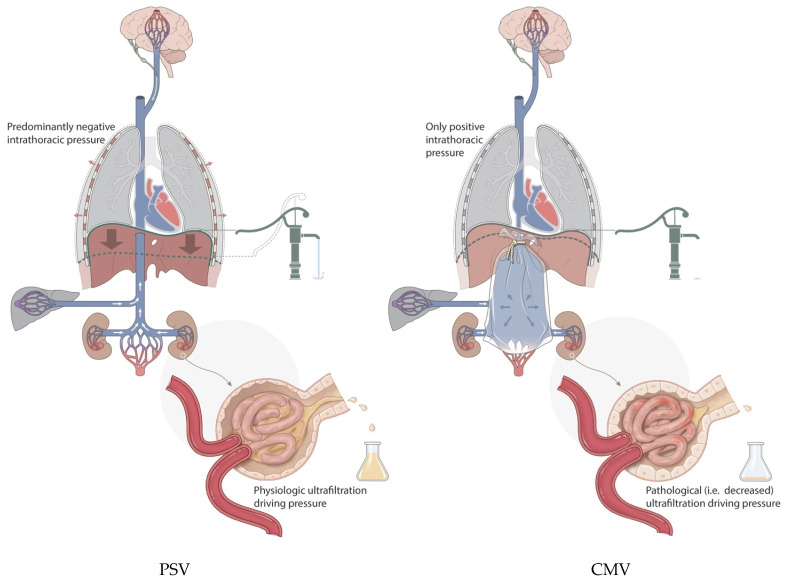
Symbolic depiction of venous bagging, i.e., venous congestion with increased venous blood volume in this compartment, showing its inherent harmful effects on the kidney, liver, and possibly brain, resulting in part from a lack of diaphragmatic pump activity under controlled mechanical ventilation (CMV; on the **right**) compared with tailored pressure support ventilation (PSV; on the **left**). Contributing factors such as the level of sedation and IV fluid tailoring are left out for clarity. Water pump in relation to diaphragm activity depicts the potential increase in venous return. With PSV, urine production is maintained compared to the occurrence of decreased kidney function when venous flow is limited with CMV.

From a clinical standpoint, renal interstitial edema appears to partly explain ICU-acquired AKI. When the intrarenal volume increases, there is no linear increase in pressure [20]. When edema occurs within the barely stretchable kidney, its hard exterior decreases the pressure gradient at the glomerular level, resulting in a reduced renal ultrafiltration gradient and clearance (see Figure 1). The positive effects of diuretics and fluid restriction on the risk of AKI support this concept [21,22]. The contribution of capillary leakage and/or venous congestion will likely vary depending on the underlying pathology and the level of intravenous resuscitation [23]. Additionally, renal ischemia resulting from edema of the renal parenchyma due to venous congestion has been proposed as a parallel mechanism [24].

## 3. Venous Congestion

The venous system plays a crucial role in returning blood from the periphery to the heart and provides capacitance to maintain heart filling [25]. Approximately 70% of the circulating volume in the cardiovascular system is held within the venous system, comprising veins, venules, and venous sinuses, which under healthy conditions can assist in increasing cardiac output when necessary [25,26,27]. An increase in venous return leads to an increase in the cardiac tidal volume and, subsequently, cardiac output, but the latter is also dependent on the sympathetic control of the heart rate. If the body does not require increased cardiac output, the heart rate will decrease with the increased tidal volume. An increase in cardiac output was seen in a study of trauma patients that revealed that the spontaneous breathing group experienced an average increase in their cardiac index of more than 1 L/min/m^2^ compared to a pressure-controlled group over a 10-day period, which is compatible with increased venous mobilization [28].

In general, the physiologic response to orthostasis aims for an increase in cardiac output. Tachycardia is obvious and an increase in diaphragm activity is also important. Inducing a temporary form of venous bagging in a healthy individual through vasoplegia as a response to warmth, hands us an easy way to visualize the importance of increased diaphragm activity. After having a healthy middle-aged man lie in lukewarm water and get up, the respiratory rate hardly changes. Having him do the same after ten minutes in hot water induces significant orthostasis, which leads to an involuntary increase in the breathing depth and frequency after standing up. In this example, the increase in breathing frequency is approximately 4–5-fold, i.e., from 6 to almost 30 times per minute (Video S1) and is the actual mobilization, i.e., pumping, of blood from the venous compartment through increased diaphragm activity. Having this pumping capacity maintained as much as possible in ventilated patients, we suggest, explains a diminished level of venous congestion and through this a diminished risk of organ failure.

During spontaneous inspiration, as opposed to CMV, there is a reduction in pleural pressure. This disparity in pressure induces an elevated differential between the mean systemic filling pressure (MSFP) and that found in the right atrium, leading to increased right heart filling [29]. The resulting effect is an increased right ventricular stroke volume during the inspiratory phase. However, the immediate consequence for the left heart is limited and is explained by the buffering capacity of the pulmonary vasculature [30]. Additionally, in young individuals, sinus arrhythmia can further increase the heart rate during inspiration and decrease it during expiration, which assists in increased venous return.

When active muscle contraction is used during expiration to increase abdominal pressure, venous return to the right heart can be further augmented. However, excessive forced expiration, such as with inadequate sedation during PSV, can be harmful by shifting the diaphragm cranially, reducing lung volume and oxygenation, and possibly causing lung injury [31].

Venous capacitance is divided into stressed and unstressed volumes. The unstressed volume does not increase the vein wall pressure, while filling above this level results in wall stretch and an inherent increase in the wall pressure, referred to as the stressed volume. The MSFP, which depends on venous recoil and not cardiac function, shapes cardiac preload through “veno-ventricular coupling”; this is along with the mechanical effects observed during spontaneous breathing efforts [32,33]. Propofol, when administered within the therapeutic range, has been shown to decrease the MSFP and, as such, increases the unstressed volume and contributes to venous congestion [34]. This is further increased when coupled with the reduced sympathetic activity caused by opioids.

The increased incidence of AKI in patients whose fluid administration is not restricted, and in those who have subsequent increased subdiaphragmatic venous pressure together with a lack of sufficiently negative pleural pressures, becomes explainable (Figure 1).

To better comprehend the key factors at play, an ideal approach would involve studying ICU patients without cardiac insufficiency or nephrotoxic factors to evaluate the consequences of venous congestion. Another method would be to induce selective abdominal venous congestion in a rat model by tying a surgical wire around the inferior vena cava (IVC), as demonstrated by Cops et al. [35,36]. Importantly, throughout this procedure, normal cardiac function and cardiac preload are maintained.

Induced renal congestion and congestive hepatopathy have been found to lead to organ damage, with specific pathological changes observed during autopsy. The organ weight, plasma creatinine, plasma cystatin, urinary albumin, glomerular surface area, and the width of Bowman’s space were all significantly increased. Additionally, inflammation and collagen deposition with hepatic fibrosis were observed, together with the same organ damage, as reported by Dong et al. [37]. These findings suggest that the increase in abdominal venous capacitance itself is the main reason for venous congestion. Additionally, it is important to note that, in the first 2 days after surgery in the rat model, the experimental group demonstrated tachypnea and hyperpnea, indicating an effort to restore venous return and thus increase cardiac output. In ICU patients, hepatic failure is less common and can be explained by the liver’s more apical position in the abdomen in relation to the bagging of venous capacitance. To effectively induce the same harm as that observed in the rat model, venous congestion in ICU patients would likely need to be more severe than what typically occurs.

By means of ultrasound, efforts have been made to visualize and grade venous congestion. This is mostly achieved by interpreting vena cava collapse and diameter and interpreting the degree of dilatation in hepatic veins. So-called venous excess ultrasound, or VExUS, includes Doppler signals of the hepatic vein, the vena portae, and the intrarenal blood flow and may help to distinguish patients at risk [38,39]. Intrarenal venous flow has been similarly quantified by the renal venous stasis index (RVSI), defined as the duration of absent venous flow divided by the duration of the cardiac cycle in patients with acute decompensated heart failure [40]. Even renal edema with the accumulation of fluid directly under the hard kidney capsule can sometimes be seen.

## 4. Pros and Cons of PSV

Mauri et al. used the double-edged sword metaphor when describing the risks and advantages of PSV [41]. PSV offers several advantages, such as improved patient comfort and reduced sedation requirements. Further favoring PSV are studies demonstrating active diaphragm contraction and reduced atrophy, improved ventilation/perfusion matching, reduced time on the ventilator, and hemodynamic improvements [42,43]. Another relevant factor is the effects of variable stretch observed when the tidal volume and frequency are varied to approximate natural variability, such as that inherent in PSV, which can enhance surfactant secretion and possibly reduce the risk of VILI [44]. Related to this, the reduced incidence of atelectasis in dorsal lung regions where diaphragmatic activity is preserved may play a pivotal role [45,46].

The prevalence of PSV as the primary ventilation mode for patients undergoing prolonged ventilation ranged from 40.5% in a study conducted in Australia and New Zealand [47] to as low as 6.4% in the SAPS 3 ventilation cohort, which included 13,322 patients admitted to 299 ICUs across 35 countries [48]. Notably, within the initial 24 h period following the transition to PSV, there was an observed augmentation in the minute ventilation and the absolute tidal volume, a phenomenon that aligns with the described mobilization of the venous volume.

Moreover, PSV can lead to increased work of breathing (WOB) and to respiratory muscle fatigue, especially when used inappropriately or with inappropriate levels of support. Hyperventilation in spontaneously breathing sheep was found to lead to acute respiratory failure due to surfactant dysfunction and noncardiogenic pulmonary edema [49]. These are important reasons not to use PSV in patients in the early phase of critical illness if they require an elevated respiratory minute volume when adequately sedated and dosed with opioids. It is obvious that PSV requires a sufficiently intact nervous system, and it is clearly not the immediate mode for patients with central apneas or other neurologic impediments prohibiting autonomous breathing efforts. Additionally, sufficient respiratory muscle strength is a requirement and this is the case in most critically ill patients, at least in the first week of their admittance to the ICU.

In recent years, techniques such as measuring the WOB and P01 values (the pressure level required to generate an inspiratory flow rate of 0.1 L per second during an occluded inspiratory effort) have gained attention in mechanical ventilation, especially as markers of air hunger and as methods for monitoring the potential harm of PSV [50]; these can assist in distinguishing specific situations where PSV might not be the most suitable mode. Furthermore, in cases where trigger settings are difficult, there is a chance that inadequate ventilation will occur due to patient–ventilator asynchrony, which can result in hypoventilation or breath stacking. This can be particularly problematic in patients with an unstable respiratory drive or compromised respiratory muscles, resulting in ineffective efforts.

## 5. Prerequisites for Safe PSV

Inadequate sedation and analgesia can exacerbate ARDS by increasing patient agitation and causing ventilator dyssynchrony. Conversely, excessive sedation can impair spontaneous breathing efforts and delay recovery. Dyspnea in ventilated patients occurs frequently and can be intense; it is strongly associated with anxiety [51,52] and has been shown to increase PTSD [53]. Appropriate sedation and opioid management are prerequisites to the optimization of ventilator settings, which have been shown to reduce the duration of mechanical ventilation [54]. Ventilator settings that leave no room for natural respiratory fluctuations can aggravate dyspnea and induce patient–ventilator dyssynchrony.

On the other hand, by aiming for complete pressure support breathing, i.e., with no additional ventilator-assisted breaths, patient–ventilator dyssynchrony can more easily be circumvented. The potential harm caused by ventilator dyssynchrony is real, and a direct association with mortality has been described in the literature [55]. It is estimated that at least one-third of patients experience frequent dyssynchrony during mechanical ventilation [56], and this can understandably be a deterrent for reducing patient sedation.

A high respiratory drive can compromise safe spontaneous breathing during assisted ventilation by inducing high lung stress and repeated vigorous inspiratory efforts, and this should be prevented [57]. Measurements such as the end-expiratory occlusion maneuver can be used to detect excessive inspiratory effort and dynamic lung stress [58]. In some patients, a moderate decrease in PaO_2_ can increase respiratory drive, and it is known that this effect becomes much stronger below 60 mmHg [59,60].

When patients are ventilated with a supportive modus, it appears that the severity of lung disease determines the increased respiratory drive and that the sedation strategy does not, notably based on the RASS score. Higher PEEP levels could then actually improve the respiratory drive [61]. Additionally, higher PEEP levels will often increase the need for more sedation. Setting the level at 12 or higher generally requires an increased dose of opioids, and possibly sedatives, due to the imposed discomfort inherent to hyperinflation.

To minimize excessive inspiratory efforts and the associated risk of lung injury, it is crucial to continually adjust the opioid and sedation levels appropriately. Achieving the desired end-tidal carbon dioxide can be accomplished by titrating medications based on individual patient blood gas analyses. Additionally, it is advisable to maintain the patient’s breathing rate within a range of 16 to 20 breaths per minute, with an upper limit of 24 breaths per minute whenever possible. This approach is generally feasible unless the patient is in a hypercatabolic state, such as during the initial phase of sepsis.

Once the optimal level of inspiratory effort has been established, it is important to periodically reduce the level of ventilatory support as the patient’s condition improves. This step is crucial to minimize the inherent risks associated with elevated pressures and hyperinflation. We have observed that, as a general guideline, it is usually not necessary to lower pressure support below 8 cm H_2_O in the period leading up to extubation when muscle strength has been preserved by the early initiation of pressure support ventilation (PSV).

Furthermore, in patients with higher-than-average organ failure, such as those requiring ECMO, it is also feasible to aim for supportive ventilation [62].

When dosing the medication and adjusting the ventilator settings, it is crucial to understand the differences between patients and their specific causes of discomfort.

## 6. The Brain and Delirium

Studies have shown that cognitive and psychiatric morbidities can be persistent in survivors of critical illness, and that conservative fluid management can have a positive influence [63,64,65,66]. Furthermore, lowering intrathoracic pressure reduces intracranial pressure [6]. A factor that has not yet been evaluated is that cerebral spinal fluid drainage to cervical lymph nodes and waste clearance by meningeal lymphatic vessels [67,68,69] may be facilitated by the increased negative intrathoracic pressures accomplished with PSV.

The convergence of deep lymphatic vessels from the cranial region toward the jugular lymphatic trunk, in combination with the thoracic duct, culminates in drainage into the venous system. This venous return occurs posterior to the left internal jugular vein at its confluence with the left subclavian vein, commonly referred to as the left venous angle [70]. Under conditions where lymphatic vessels face impedance in drainage, they undergo dilatation. This phenomenon, akin to peripheral lymphedema, is also observed in meningeal lymphatic vessels.

We suggest that inhibited flow within the meningeal lymphatic vessels, stemming from the lack of effective suction caused by insufficient negative pleural pressure, or the lack of this as with CMV, and the inherent stasis of waste, may be a factor contributing to the occurrence of ICU delirium. While a direct study illustrating this is lacking, it stands as a hypothesis-generating notion. Conceivably, a physiological basis may well exist by which the augmented drainage of the cerebral lymph during spontaneous respiration can be connected to the more efficacious elimination of stagnant waste from the cranial milieu.

## 7. Conclusions and the Future

The growing interest in personalized medicine and the early adoption of PSV for eligible patients indicate potential for improving patient outcomes. Discovering the additional advantages of early PSV could lead to the further refinement of patient care, prompting the ongoing evolution of criteria for its early implementation. Nevertheless, to gain a comprehensive understanding of both the benefits and potential risks associated with early PSV, additional research is imperative. Ultrasound in the ICU may increasingly prove to be a useful tool in distinguishing patients at risk of venous congestion.

In the realm of critical care, the more assertive utilization of supportive ventilation and physiological factors that can enhance venous return, cardiac filling and inherent surfactant production will likely contribute to the mitigation of organ failure.

To optimize patient outcomes, it is crucial to conduct prospective trials that examine the impact of both controlled mechanical ventilation and pressure support ventilation on renal function. Additionally, studies focused on renal function should consider the mode of ventilation. Furthermore, research should address the relationship between delirium and PSV in critically ill patients.

In conclusion, through continued investigation into the potential benefits of early PSV and its influence on various physiological factors, we believe we can unlock new opportunities to enhance patient care by reducing the incidence of organ failure in critical care settings.

## Figures and Tables

**Table 1:** Factors to consider in acute kidney injury (AKI) among ICU patients. HUS, hemolytic uremic syndrome; TTP, thrombotic thrombocytopenic purpura; SLE, systemic lupus erythematosus; VILI, ventilator-induced lung injury; VIKI, ventilator-induced kidney injury.

Prerenal	Examples
Shock:	
- Diminished cardiac output	adrenal insufficiency, heart failure
- Hypovolemia	
- Vasoplegia	sepsis, sedation/opioids
Vascular obstruction	vasculitis
Hypoxia	asphyxia
Renal	
Renal interstitial edema	
Ischemia	
Toxic injury	medication, rhabdomyolysis, sepsis
Vascular	vasculitis, HUS/TTP, sickle cell crisis, SLE
Nephritis	radiation
Infection	
Hemolysis	
Osmotic	
Infiltrative	amyloidosis, lymphomatous
Postrenal urinary	
	prostatic hyperplasia, kidney stones, retroperitoneal fibrosis, bladder dysfunction
Postrenal venous	
Abdominal compartment syndrome	renal interstitial edema
Venous bagging	renal interstitial edema
- Exacerbation by high PEEP	
- Predominant controlled mechanical ventilation lacking decreased pleural pressures	
Miscellaneous	
Cytokines, proinflammatory mediators	VILI/VIKI, sepsis
Hypercapnia	
Permissive hypoxemia	
Genetic/metabolic	

## Data Availability

The data are contained within the article.

## Supplementary Materials

The following supporting information can be viewed at: https://jumpshare.com/v/WyBjckEmIpfKAVRYP7tu, Video S1: Venous Bagging in Hot Bathwater Experiment.
